# Macroalgae Specialized Metabolites: Evidence for Their Anti-Inflammatory Health Benefits

**DOI:** 10.3390/md20120789

**Published:** 2022-12-19

**Authors:** Djenisa H. A. Rocha, Diana C. G. A. Pinto, Artur M. S. Silva

**Affiliations:** LAQV-REQUIMTE & Department of Chemistry, University of Aveiro, 3810-193 Aveiro, Portugal

**Keywords:** anti-inflammatory, seaweed, specialized metabolites, phlorotannins, bromophenols, chromenes, terpenoids, fucoxanthin, fucosterol, caulerpin, fatty acids

## Abstract

Inflammation is an organism’s response to chemical or physical injury. It is split into acute and chronic inflammation and is the last, most significant cause of death worldwide. Nowadays, according to the World Health Organization (WHO), the greatest threat to human health is chronic disease. Worldwide, three out of five people die from chronic inflammatory diseases such as stroke, chronic respiratory diseases, heart disorders, and cancer. Nowadays, anti-inflammatory drugs (steroidal and non-steroidal, enzyme inhibitors that are essential in the inflammatory process, and receptor antagonists, among others) have been considered as promising treatments to be explored. However, there remains a significant proportion of patients who show poor or incomplete responses to these treatments or experience associated severe side effects. Seaweeds represent a valuable resource of bioactive compounds associated with anti-inflammatory effects and offer great potential for the development of new anti-inflammatory drugs. This review presents an overview of specialized metabolites isolated from seaweeds with in situ and in vivo anti-inflammatory properties. Phlorotannins, carotenoids, sterols, alkaloids, and polyunsaturated fatty acids present significant anti-inflammatory effects given that some of them are involved directly or indirectly in several inflammatory pathways. The majority of the isolated compounds inhibit the pro-inflammatory mediators/cytokines. Studies have suggested an excellent selectivity of chromene nucleus towards inducible pro-inflammatory COX-2 than its constitutive isoform COX-1. Additional research is needed to understand the mechanisms of action of seaweed’s compounds in inflammation, given the production of sustainable and healthier anti-inflammatory agents.

## 1. Introduction

The seaweeds or macroalgae belong to the basic tropic level in the marine water ecosystem and are responsible, with microalgae, for the balance of the abiotic and biotic factors of marine life, either directly or indirectly [1]. Seaweeds reside in the littoral zone and are considered the oceans’ principal resource in terms of economic and ecological significance [2]. Food and Agriculture Organization (FAO) data state that global seaweed output (aquaculture and wild) has increased from 2000 to 2019 nearly threefold, from 118,000 tons to 358,200 tons [3]. The world’s seaweed production mainly comes from the five major continents, with Europe accounting for 0.8% of global seaweed production. In Europe, 96% of seaweed is naturally obtained, with 2010 marking the start of its cultivation. Continental Portugal and its two archipelagos (the Azores and Madeira Islands) present an exciting and diverse seaweed community mainly due to the latitudinal gradients, coast length, and climate conditions. Although Portuguese seaweeds have been relatively underexplored in terms of their economic benefits, in recent years, they are being used in various applications such as cosmetics, commercial harvest, and pharmaceuticals [4]. Many Portuguese institutions of higher education have research groups dedicated to seaweed studies which contribute to a better understanding of their properties, potential, and applications [5,6,7,8,9].

Natural pigments come in various colours and have been broadly used since ancient times, and more recently, are highly valued in biotechnology and pharmacology. The seaweed colour is determined by the pigment abundance, and their classification into one of the three main phyla (red (Rhodophyceae), brown (Phaeophyceae), and green (Chlorophyceae)) is also associated with the pigments produced. Brown seaweeds are extensive and can range from giant kelp (20 m long) to thick, leather-like seaweeds (2–4 m long) to smaller species (30–60 cm long), and in terms of pigments, present high concentrations of fucoxanthin, chlorophyll a and c, and β-carotene. Red seaweeds are smaller than the other seaweeds, ranging from a few centimetres to about a meter in length, with high amounts of phycoerythrin and phycocyanin. Green seaweeds are also smaller than brown seaweeds, with a size similar to red seaweeds, and they have a high content of chlorophylls a and b [7,10,11].

Seaweed and its products are particularly low in calories but rich in primary metabolites such as vitamins, minerals, proteins, and polysaccharides. Another class of compounds produced by algae tissues are the specialized metabolites, mainly phenolic compounds, including halogenated ones, sterols, terpenes, and mycosporine-like amino acids [12,13,14]. It is known that incorporating seaweed into the human diet is linked to a lower risk of a range of syndromes linked to inflammation, such as diabetes, cancer, cardiovascular disease, and obesity [2,15]. Another important use of seaweed or seaweed-based products is in cosmetics, where they can substitute equivalent synthetic products. The application of seaweed in the cosmetic industry is based on its bioactive compounds, primarily carbohydrates and proteins, but also some specialized metabolites, such as phenolic compounds, fatty acids, and terpenoids. Seaweeds are used due to the aforementioned biologically active ingredients and because they supply organic dyes, texturing stabilizers or emulsifiers, and various exciting molecules that can be applied in skincare. Moreover, seaweeds are photosynthetic organisms that produce specialized metabolites which protect the cell mechanisms and organelles and are used in sunscreens as photo-protective ingredients [16,17].

Chronic inflammation is characterized by the continuous sending of inflammatory cells, even when there is no outside danger, which leads to persistent host tissue damage. As a result, different pathologies such as cardiovascular disease (CVD), atherosclerosis, inflammatory bowel disease (IBD), multiple sclerosis, rheumatoid arthritis, and neurodegeneration [18] can upraise. Currently, the medications used for pain and inflammation are food supplements (vitamins A, C, D, and zinc), nonsteroidal anti-inflammatory drugs (NSAID), and steroid injections. Most NSAID, such as aspirin, indomethacin, ibuprofen, ketoprofen, flurbiprofen, and diclofenac, contain carboxylic groups. These can alleviate pain and inflammation by blocking the metabolism of arachidonic acid by inhibiting cyclooxygenase enzyme and reducing prostaglandin production. Unfortunately, NSAID’s excellent anti-inflammatory potential is accompanied by severe side effects such as gastrointestinal ulceration, perforation, obstruction, and bleeding. Steroid injections such as corticosteroid shots should not be used more than three or four times in the same body part per year [19,20]. So, encouraging researchers to explore alternative templates with an excellent anti-inflammatory potential and satisfactory tolerability, in addition to an effective long-term strategy to fight against inflammation-associated disorders, is imperative.

One of the most widespread chronic inflammatory diseases is rheumatoid arthritis (RA). Its prevalence ranges from 0.5% to 2% in the general population, particularly in women and smokers, who are often affected, and is considered a disease with some genetic prevalence. Genetics, autoimmunity, and environmental factors may play a pathogenic role in the disease, although it has not yet been fully elucidated. The joints are primarily affected, but it should be considered that inflammation includes extra-articular manifestations, such as pulmonary involvement or vasculitis, and systemic comorbidities. In the past decade, we have noticed some therapeutic improvements in treating rheumatoid arthritis that has transformed articular and systemic outcomes. However, treatment for rheumatoid arthritis is still needed [21,22].

Macroalgae offer an appreciated source of chemical compounds with anti-inflammatory potential and with few or no side effects [23,24]. Anti-inflammatory compounds can be found in marine species, and macroalgae are an excellent source of these biocompounds [25], from which fucoidans and galactofucan can be highlighted [26]. It is worth mentioning that use of seaweeds in food and traditional medicine has been recorded in early archaeological data, especially in many Asian countries like China, Indonesia, Korea, Japan, and Malaysia [27]. The ethnopharmacological significance in traditional Chinese medicine should be highlighted because seaweeds are used to treat many inflammatory diseases, from oedema, furuncles, and haemorrhoids to cardiovascular diseases [28].

Mitogen-activated protein kinase (MAPK) and nuclear factor-kappa B (NF-κB) signalling play a vital role in inflammatory processes. The signalling pathway complexity includes the production of nitric oxide (NO) and PGE2 and the expression of inflammation-related enzymes, such as COX-2 and inducible nitric oxide synthase (iNOS). NO is an endogenous free radical and a pro-inflammatory mediator of the cytokines TNF-α, IL-6, and IL-1β expression and production. ILs and TNF-α are involved in initiating the inflammatory pathways involved in the atrioventricular node (AVN), to cite one example. Inflammation markers such as the aforementioned can activate macrophages that will induce the release of pro-inflammatory cytokines. Interleukins induce the synthesis of acute-phase proteins, and TNF-α is one of the essential pro-inflammatory cytokines that participates in vasodilatation, oedema formation, and leukocyte adhesion. These phenomena are attributed to phosphorylation and the activation of the signal transduction factors (extracellular signal-regulated kinase (ERK), I-κB kinase (IKK), c-Jun *N*-terminal kinase (JNK), and MAPK), and the expression of transcription factors (inhibitor of NF-κB (I-κB) and NF-κB) [29,30,31,32].

## 2. Specialized Metabolites with Anti-Inflammatory Activity

Macroalgae use is increasing and spreading. What was a common food ingredient in Oriental cuisine is nowadays an additive in several smart foods and folk medicine formulations. Some formulations are sold as promoters of health benefits, including anti-inflammatory effects. Although several macroalgae extracts, as stated above, showed anti-inflammatory activity, their specialized metabolites must also be tested, and their amount in the formulations must be established. Moreover, in vivo studies and clinical trials are still required to validate the claimed potential in pharmaceutical formulations. Knowing the limitations of the in vitro studies, several authors are moving forward and focusing their biological assays using in vivo models. Unfortunately, anti-inflammatory clinical trials are still needed. This literature survey aims to give a critical synopsis of the current state of the art regarding the anti-inflammatory effects of essential macroalgae specialized metabolites, emphasizing their molecular mechanisms. The following sections will present and discuss specific examples chosen by the authors and consider the most promising anti-inflammatory compounds, as well as the ones for which the studies are broadening, including mechanism of action, and, if possible, in vivo studies.

### 2.1. Phlorotannins

**Figure 1:**
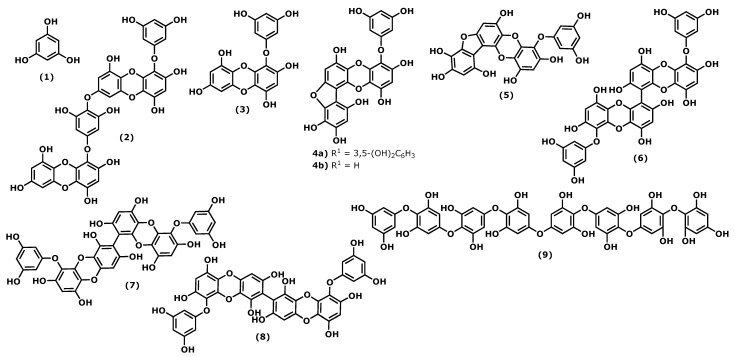
Structures of the phlorotannins whose anti-inflammatory activity is discussed.

Phlorotannins are polymers of benzene-1,3,5-triol (**1**), commonly named phloroglucinol (Figure 1). The polymerization occurs through a single ether bond, 1,4-dibenzodioxin linkage, or by direct covalent bond between the benzene rings [11,33]. Phlorotannins have a wider mass range (from 125 to 1 × 105 Da or higher) and different structures. They can be divided into four classes: fuhalols and phlorethols (ether bond), fucols (phenyl bond), fucophloroethols (ether and phenyl bonds), and eckols and carmalols (a dibenzodioxin bond). They are found in high quantities in brown algae and are considered the specialized metabolites responsible for the pharmacological activities of some *Ecklonia* and *Eisenia* species, such as *Ecklonia cava*, Kjellman, 1885 [34,35]. Phlorotannins have demonstrated their anti-inflammatory effects by inhibiting hyaluronidase, phospholipase A2, lipoxygenase, and cyclooxygenase (COX) enzymes, which are involved in the inflammatory response, as well as chemical mediators and pro-inflammatory cytokines [36,37,38,39].

Considering the phloroglucinol (**1**) (Figure 1) anti-inflammatory effects, it is worth mentioning that inhibitory effects on oxidative stress were reported, and phloroglucinol (**1**) is also able to inhibit the production of tumour necrosis factor-α (TNF)-α, interleukin-1β, interleukin-6 ((IL)-1β), and IL-6, and prostaglandin E(2) (PGE2) in lipopolysaccharide (LPS)-stimulated RAW264.7 cells. Additionally, phloroglucinol decreased the expression of matrix metalloproteinases (MMPs) in the human fibrosarcoma cell line HT1080. MMPs are known to be involved in several inflammatory conditions. Finally, phloroglucinol inactivates the NF-κB-inducing kinase (NIK) and the kinases ERK and MAPK, preventing inflammation episodes [40].

Dieckol (4-(4-((6-(3,5-dihydroxyphenoxy)-4,7,9-trihydroxydibenzo[*b*,*e*][1,4]dioxin-2-yl)oxy)-3,5-dihydroxyphenoxy)dibenzo[*b*,*e*][1,4]dioxine-1,3,6,8-tetraol (**2**)), phlorofucofuroeckol(PFF) B (4-(3,5-dihydroxy-phenoxy)benzo[*b*]benzo[5,6][1,4]dioxino[2,3-*e*]benzofuran-1,3,6,9,10,12-hexaol (**4b**)), and fucofuroeckol-A (4-(3,5-dihydroxyphenoxy)benzo[*b*]benzo[5,6][1,4]dioxino[2,3-*e*]benzofuran-1,3,6,9,10,12-hexaol (**5**)) (Figure 1) suppresses lipopolysaccharide (LPS)-induced production of NO, PGE2 and expression of pro-inflammatory proteins (nitric oxide synthase (iNOS), COX-2, tumour necrosis factor (TNF)-α and interleukin (IL)-1β, and IL-6) in a dose-dependent manner in RAW 264.7 macrophages and BV2 microglia cells. Normally, the inhibition profile of these compounds is associated with their ability to inhibit NF-κB and p38 mitogen-activated protein kinases (MAPKs) activation [30,37,39,41].

In a comparative study of the effects of dieckol (**2**) and eckol (4-(3,5-dihydroxyphenoxy)dibenzo[*b*,*e*][1,4]dioxine-1,3,6,8-tetraol (**3**)) (Figure 1) on LPS-mediated hyperpermeability and monocytes migration in human umbilical vein endothelial cells (HUVECs), it was observed that dieckol (**2**) has a better inhibitory effect than eckol (**3**), probably due to the higher number of hydroxy groups in the dimeric structure [42]. However, Eom et al. [43] showed that eckol (**3**) could inhibit the expression of various inflammatory cytokines in *Propionibacterium acnes*-induced human skin keratinocytes (HaCaT) cells. It inhibited the expression levels of MMPs (MMP-2 and -9), inflammatory mediators in a concentration-dependent manner, acting at the transcriptional level. Its anti-inflammatory properties were associated with the inhibition of p-NF-κB p65 and p-Akt at the translational level [43].

In the last decade, it was demonstrated that 6,6′-bieckol (6,6′-bis(3,5-dihydroxyphenoxy)-[1,1′-bidibenzo[*b*,*e*][1,4]dioxin]-2,2′,4,4′,7,7′,9,9′-octaol (**6**)) (Figure 1), isolated from *E. cava*, inhibited the expression and release of NO, PGE2, TNF-α, and IL-6 in LPS-stimulated macrophages, with concomitant inhibition of NF-κB activation [44]. More recently, Sugiura et al. [45] demonstrated using a rat mast cell line (RBL-2H3) that 6,6′-bieckol (**6**) and other phlorotannins such as eckol (**3**), 6,8′-bieckol (6,9′-bis(3,5-dihydroxyphenoxy)-[1,2′-bidibenzo[*b*,*e*][1,4]dioxin]-1′,2,3′,4,6′,7,8′,9-octaol (**7**)), 8,8′-bieckol (9,9′-bis(3,5-dihydroxyphenoxy)-[2,2′-bidibenzo[*b*,*e*][1,4]dioxin]-1,1′,3,3′,6,6′,8,8′-octaol (**8**)), PFF-A (4,9-bis(3,5-dihydroxyphenoxy)benzo[*b*]benzo[5,6][1,4]dioxino[2,3-*e*]benzofuran-1,3,6,10,12-pentaol (**4a**)), and PFF-B (**4b**) isolated from *Eisenia arborea*, Areschoug, 1876, suppressed the release of chemical mediators (histamine, leukotriene B4, and PGE2) COX-2 mRNA expression and inhibited COX-2 activity at a 500 μM of concentration [45].

Octaphlorethol A (2-(4-(4-(4-(4-(4-(4-(3,5-dihydroxyphenoxy)-3,5-dihydroxyphenoxy)-3,5-dihydroxyphenoxy)-3,5-dihydroxyphenoxy)-2,6-dihydroxyphenoxy)-2,6-dihydroxyphenoxy)-2,6-dihydroxyphenoxy)benzene-1,3,5-triol (**9**)) can be isolated from the brown marine alga *Ishige foliacea*, Okamura, 1936, and presents anti-inflammatory potential by inhibiting the CpG-stimulated primary murine bone marrow-derived macrophages and dendritic cells. The pre-treatment with octaphlorethol A (**9**) caused strong inhibition of IL-12 p40, IL-6 and TNF-α production, indicating the inhibitory effect of this compound on pro-inflammatory cytokine production. It also demonstrated the inhibitory effect on TLR9-dependent MAPK and NF-ƘB activation [46].

In terms of in vivo assays, as far as we could find, they can be considered scarce, and more studies are needed; moreover, several aspects of the phlorotannins’ mechanisms of action need to be clarified. The leukocyte adhesion of endothelial cells and transendothelial migration (TEM) of leukocytes are essential steps in the pro-inflammatory response. In in vivo assays, both compounds (**2**) and (**3**) exhibited an effectively inhibitory effect on the leakage of dye into the peritoneum in mice and decreased leukocytes count at a dose of 10 μΜ of concentration [47].

The phlorotannins eckol (**3**), 6,6′-bieckol (**5**), 6,8′-bieckol (**6**), 8,8′-bieckol (**7**), PFF-A (**4a**), and PFF-B (**4b**) were administrated orally to mice, which were previously injured using arachidonic acid (AA), 12-*O*-tetradecanoylphorbol-13-acetate (TPA) and oxazolone (OXA), and proved to be able to suppress the AA, TPA, and OXA-induced mouse ear swelling. Moreover, their positive effect was better than the epigallocatechin gallate (EGCG), which was used as the positive control influence. The 6,8′-bieckol at 75 nmol exhibited the most potent suppression (77.8%) compared with the epigallocatechin gallate’s weakest suppression (5.7%) [44].

Phlorotannins are one of the most studied macroalgae-derived metabolites; nevertheless, their potential use as new anti-inflammatory drugs needs additional studies, such as pharmacokinetic and clinical trials.

### 2.2. Bromophenols

Bromophenols are phenols bearing bromine and hydroxy groups in one or more benzene rings and are amongst the specialized metabolites produced by macroalgae [48]. Actually, bromophenols are ubiquitous in the three types of macroalgae [49,50], although they were first found in red *Rhodomela larix* (Turner), C. Agardh, 1822, (the current accepted name is *Neorhodomela larix* (Turner), Masuda, 1982) [51]. Several pharmaceutical potentials have been reported for these natural compounds [50]; however, their anti-inflammatory properties were scarcely explored.

**Figure 2:**
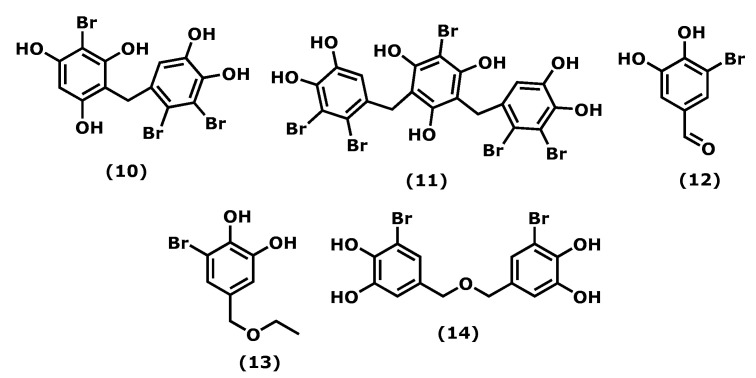
Structures of the bromophenols whose anti-inflammatory activity is discussed.

Vidalol A and B (2-bromo-4-(2,3-dibromo-4,5-dihydroxybenzyl)benzene-1,3,5-triol (**10**) and 2-bromo-4,6-bis(2,3-dibromo-4,5-dihydroxybenzyl)benzene-1,3,5-triol (**11**)) (Figure 2), isolated from the red algae *Vidalia obtusiloba* (Mertens ex C. Agardh), J. Agardh, 1863, are examples of compounds that inhibit the bee venom-derived phospholipase A2 (PLA2), showing 96% enzyme inactivation at 1.6 μg/mL. An in vivo assay in the phorbol ester (PMA)-induced swelling mouse ear showed that (**10**) and (**11**) reduced oedema (58–82%) significantly when applied topically [52].

The 3-bromo-4,5-dihydroxybenzaldehyde (3-BDB) (**12**) (Figure 2) is another example of a bromophenol (isolated from marine red algae, such as *Polysiphonia morrowii*, Harvey, 1857, *Polysiphonia urceolata* (Lightfoot ex Dillwyn), Greville, 1824, and *Rhodomela confervoides* (Hudson), P. C. Silva, 1952 [53,54]) that in LPS-stimulated RAW 264.7 murine macrophages can suppress the production of IL-6, a pro-inflammatory cytokine, in a dose-dependent manner. BDB also had an inhibitory effect on the phosphorylation of nuclear factor NF-κB, a signal transducer and activator of transcription 1 (STAT1; Tyr 701), which are two major signalling molecules involved in cellular inflammation. The in vivo assay of BDB (**12**) on atopic dermatitis (AD) in BALB/c mice induced by 2,4-dinitrochlorobenzene (DNCB) showed that treatment (100 mg/kg) resulted in suppression of the development of AD symptoms compared with the control treatment. 3-BDB (**12**) also reduced immunoglobulin E levels in serum, smaller lymph nodes with reduced thickness and length, decreased ear oedema, and reduced levels of inflammatory cell infiltration in the ears [55]. With a similar structure to BDB (**12**), 3-bromo-5-(ethoxymethyl)benzene-1,2-diol (BEMB) (**13**) (Figure 2), also isolated from the red algae *P. morrowii*. BEMB (**13**) demonstrated anti-inflammatory effects by inhibiting the production of NO, the expression of iNOS, and COX-2 in the LPS-activated RAW 264.7 cells and zebrafish embryos without cytotoxicity. It suppressed the protein and mRNA expression levels of nuclear factor NF-ƘB in the LPS-activated RAW 264.7 cells and zebrafish model [56]. The last example is bromophenol bis(3-bromo-4,5-dihydroxybenzyl) ether (BBDE) (**14**) (Figure 2) isolated from the same algae, which can inhibit inflammation by reducing inflammatory mediators, such as NO, prostaglandin E2, iNOS, COX-2, and pro-inflammatory cytokines (TNF-α, IL-1β, and IL-6), in LPS-induced RAW 264.7 macrophage cells [57].

Considering the number of bromophenols found in macroalgae, their anti-inflammatory potential is scarcely studied; more toxicological and in vivo studies are needed, and, in some cases, clinical trials would be appreciated.

### 2.3. Chromenes

Chromenes or benzopyrans represent the basic nucleus of various seaweed compounds with an anti-inflammatory potential, which includes the inhibition of COX and lipoxygenase, enzymes linked to inflammatory manifestations.

**Figure 3:**
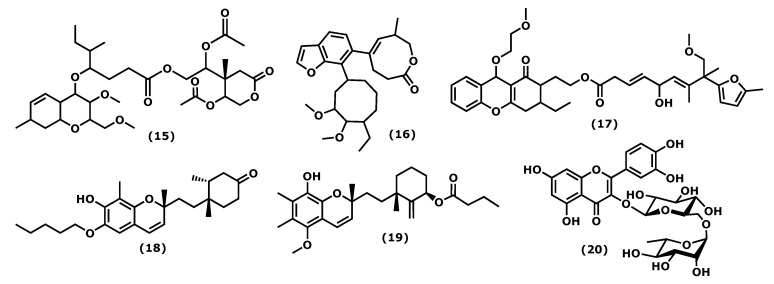
Structures of the chromene derivatives whose anti-inflammatory activity is discussed.

The 2-acetoxy-2-(5-acetoxy-4-methyl-2-oxotetrahydro-2*H*-pyran-4-yl)ethyl 4-(3-methoxy-2-methoxymethyl-7-ethyl-3,4,4a,7,8,8a-hexahydro-2*H*-chromen-4-yloxy)-5-methyl-heptanoate (**15**) (Figure 3), isolated from the red seaweed *Gracilaria opuntia*, Durairatnam, nom. Inval., 1962, showed a moderate anti-inflammatory activity against the COX-2 isoform (IC_50_ 0.96 mg/mL) than COX-1 (IC_50_ 1.21 mg/mL), in comparison to the traditional NSAID, such as aspirin (anti-COX-1 IC_50_ 0.005, anti-COX-2 IC_50_ 0.21 mg/mL) and ibuprofen (anti-COX-1 IC_50_ 0.04 mg/mL, anti-COX-2 IC_50_ 0.09 mg/mL). The in vitro 5-lipoxygenase (5-LOX) activity (IC_50_ 1.22 mg/mL) was comparable to that of synthetic ibuprofen (IC_50_ 0.93 mg/mL) [58]. Further, isolated from *G. opuntia*, the 5-[7-(5-ethyl-3,4-dimethoxycyclooctyl)benzofuran-6-yl]-7-methyl-3,4,7,8-tetrahydro-2*H*-oxocin-2-one (**16**) and 2-(3-ethyl-9-(2-methoxyethoxy)-1-oxo-2,3,4,9-tetrahydro1*H*-xanthen-2-yl)ethyl 5-hydroxy-9-methoxy-7,8-dimethyl-8-(5-methylfuran-2-yl)nona-3,6-dienoate (**17**) (Figure 3) exhibited inhibitory activities towards pro-inflammatory cyclooxygenase-2/5-lipoxygenase (COX-1, 2, and 5-LOX). Both compounds had a comparable inhibitory effect in 5 LOX (IC_50_ 0.209 × 10^−2^ M) with synthetic non-steroidal anti-inflammatory drugs (NSAID) ibuprofen (IC_50_ 0.451 × 10^−2^ M, *p* < 0.05) and selectivity towards COX inhibition (SI: anti-COX-1 IC_50_/anti-COX-2 IC_50_ ~1.08–1.09) than NSAID (aspirin, and ibuprofen, SI: 0.02 and 0.44, respectively, *p* < 0.05) [59].

4′-[10′-[7-Hydroxy-2,8-dimethyl-6-(pentyloxy)-2*H*-chromen-2-yl]ethyl]-3′,4′-dimethyl-cyclohexanone (**18**) and 3′-[10′-(8-hydroxy-5-methoxy-2,6,7-trimethyl-2*H*-chromen2-yl)ethyl]-3′-methyl-2′-methylene cyclohexyl butyrate (**19**) (Figure 3), isolated from the red seaweed *Gracilaria Salicornia* (C. Agardh), E. Y. Dawson, 1954, were tested against pro-inflammatory 5-LOX, and compound (**19**) registered significantly higher activity (IC_50_ 2.03 mM) than that displayed by (**18**) (IC_50_ 2.46 mM, *p* < 0.05). The compound selectivity index was also higher (IC_50_ anti-COX-1/IC_50_ anti-COX-2 > 0.95) than that exhibited by the non-steroidal anti-inflammatory agent ibuprofen (0.89) (*p* < 0.05). These studies suggested that chromenyls have higher selectivity towards inducible pro-inflammatory COX-2 than its constitutive isoform COX-1 [60].

Concerning phenolic compound, rutin (2-(3,4-dihydroxyphenyl)-5,7-dihydroxy-3-(((2*S*,4*S*,5*S*,6*R*)-3,4,5-trihydroxy-6-((((2*R*,3*R*,4*R*,5*R*,6*S*)-3,4,5-trihydroxy-6-methyltetrahydro-2*H*-pyran-2-yl)oxy)methyl)tetrahydro-2*H*-pyran-2-yl)oxy)-4*H*-chromen-4-one (**20**)) (Figure 3), identified in the crude extract of *Porphyra dentata*, Kjellman, 1897, (the current accepted name Neoporphyra dentata (Kjellman), L.-E. Yang and J. Brodie) inhibited NO production in LPS-stimulated RAW 264.7 cells. Its activity was compared to the obtained for catechol, and it was observed that catechol was a more potent suppressor of the up-regulation of iNOS promoter and NF-κB enhancer than rutin (**20**). Catechol (1–11 μg/mL) inhibited iNOS promoter activity to a greater extent than rutin (80–250 μg/mL) in a dose-dependent manner. Catechol (11 μg/mL) and rutin (250 μg/mL) decreased LPS-induced NF-κB enhancer activity to six- and twofold, respectively [61]. It is relevant that some of these metabolites were evaluated for their selectivity index; however, few action mechanisms were revealed, and in vivo studies are suggested.

### 2.4. Terpenoids

Terpenoid is a general term for hydrocarbons and their oxygen-containing derivatives obtained through isoprene unit polymerization. They are usually classified into monoterpenes, sesquiterpenes, diterpenes, and polyterpenes according to their structural units [62] and are recognized for their biological activities, from which anticancer can be highlighted [63].

**Figure 4:**
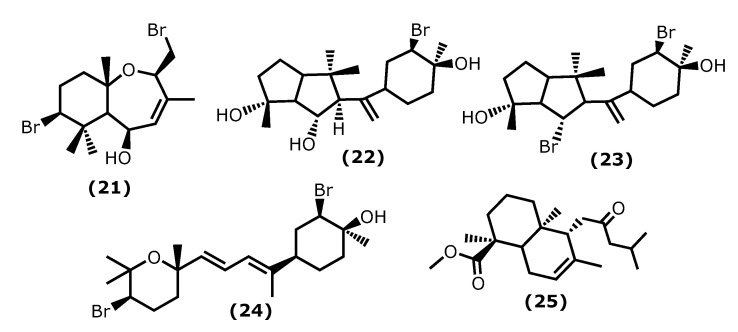
Structures of the terpenoids whose anti-inflammatory activity is discussed.

5β-Hydroxypalisadin B [(2*R*,5*R*,7*S*,9a*S*)-7-bromo-2-(bromomethyl)-3,6,6,9a-tetramethyl-2,5,5a,6,7,8,9,9a-octahydrobenzo[*b*]oxepin-5-ol (**21**)] (Figure 4), a sesquiterpene isolated from the red algae *Laurencia snackeyi* (Weber Bosse), M. Masuda, 1997, [64], suppressed the NO production, iNOS, and COX-2 expression and cytokine release in LPS-stimulated RAW 264.7 cells. Moreover, LPS-induced NO production was dose-dependently decreased with a maximum of 90% inhibition observed at the concentration of 50 µM [64]. The in vivo studies performed in lipopolysaccharide (LPS)-induced zebrafish embryo using 0.25, 0.5, and 1 μg/mL of compound (**21**) showed a profound protective effect of this compound in the zebrafish embryo as confirmed by survival and heartbeat rate, and yolk sac oedema size. It inhibited the LPS-induced NO production in a dose-dependent manner. Moreover, 5β-hydroxypalisadin B (**21**) showed a protective effect compared to dexamethasone, the standard anti-inflammatory agent [65].

Neorogioltriol ((1*R*,5*S*,6*S*)-5-(1-((3*R*,4*S*)-3-bromo-4-hydroxy-4-methylcyclohexyl)vinyl)-1,4,4-trimethyloctahydropentalene-1,6-diol (**22**)) (Figure 4) is a tricyclic diterpenoid isolated from the red algae *Laurencia glandulifera* (Kützing), Kützing, 1849, ref. [66] and was evaluated using the writhing test, showing that 1 mg/kg (b.w.) was enough to reduce the mouse acetic acid-induced writhing response by 88.9% [66]. The in vivo tests using formalin-induced licking in rats showed that compound (**22**) affected neurogenic and/or inflammatory pain. Neorogiotriol (**22**) exhibited a remarkable reduction in the licking time by 48% in the second phase, which begins at 20 min and can last up to 60 min, representing inflammatory pain. This inhibition effect obtained in the second phase of the performed test is typical of COX inhibitors, suggesting peripheral analgesic activity [66].

Neorogioldiol ((1*R*,6*S*)-6-bromo-5-(1-((3*R*,4*S*)-3-bromo-4-hydroxy-4-methylcyclohexyl)vinyl)-1,4,4-trimethyloctahydropentalen-1-ol (**23**)) and *O*^11^,15-cyclo-14-bromo-14,15-dihydrorogiol-3,11-diol (**24**) (Figure 4) are two brominated diterpenoids found in *Laurencia* sp., and, together with compound (**22**), were assessed for their anti-inflammatory capacity in vitro using RAW 264.7 cells [67]. Compounds (**23**) and (**24**) were also evaluated in vivo using C57BL/6J mice with dextran sodium sulphate (DSS)-induced inflammatory bowel disease (colitis) [67]. All compounds (**22**–**24**) suppress macrophage activation and promote an M2-like anti-inflammatory phenotype by inducing expression of arginase 1, MRC1, IRAK-M, the transcription factor C/EBPβ, and the miRNA miR-146a; also, they suppressed iNOS induction and NO production [67]. The C57BL/6J mice received 2.5% DSS in their drinking water and were injected intraperitoneally with compounds (**23**) and (**24**) every second day for 5 days. All DSS-treated mice showed a reduction in colon length, which confirms colonic inflammation macroscopically, as well as a very significant decrease in pro-inflammatory cytokine messenger RNA (mRNA) (more than a 40-fold decrease in the case of interleukin-6) [67].

Lastly, the diterpenoid methyl 16(13→14)-abeo-7-labdebe(12-oxo)carboxylate (**25**) (Figure 4) isolated from the red algae *G. salicornia* presented a similar anti-inflammatory effect against pro-inflammatory 5-LOX (IC_50_ 0.86 mg/mL) comparative to the ibuprofen (IC_50_ 0.92 mg/mL, *p* < 0.005) [68].

### 2.5. Fucoxanthin

**Figure 5:**
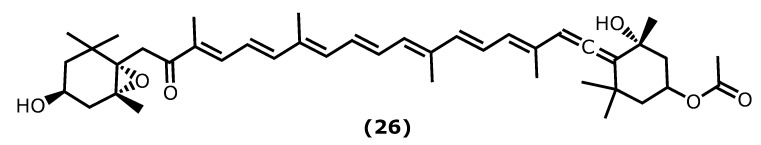
Fucoxanthin structure.

Fucoxanthin ((3*R*)-3-hydroxy-4-((3*E*,5*E*,7*E*,9*E*,11*E*,13*E*,15*E*)-18-((1*S*,4*S*,6*R*)-4-hydroxy-2,2,6-trimethyl-7-oxabicyclo[4.1.0]heptan-1-yl)-3,7,12,16-tetramethyl-17-oxooctadeca-1,3,5,7,9,11,13,15-octaen-1-ylidene)-3,5,5-trimethylcyclohexyl acetate (**26**)) (Figure 5) is the most abundant natural carotenoid, accounting for approximately 10% of nature’s carotenoids. It is found mainly in brown algae and structurally contains allene bonds, 5,6-monocyclic oxide, and acetylated groups. Beneficial health effects have been reported for fucoxanthin (**26**), the reason why it is one of the most studied metabolites [69]. Regarding the anti-inflammatory effect, the literature survey indicates that fucoxanthin has a protective effect on various inflammation-related diseases. From which diabetes [70,71], neurodegenerative [72,73,74], skin and liver [75,76], inflammatory pain [77], and cardiovascular [78,79] can be highlighted.

Recent studies showed that fucoxanthin has a significant pharmacological effect on diseases related to oxidative stress injury. Its mechanism of action is primarily related to nuclear factor-erythroid 2-related (Nrf2) signal transduction pathway and gut microbiota regulation [80]. Zheng et al. [81] showed that fucoxanthin increased the phosphorylation level of the Akt/Nrf2 pathway as well as its effect on increased the mRNA and proteins levels of glutamate-cysteine ligase catalytic subunit (GCLC) and glutathione synthetase (GSS) in human keratinocytes (HaCaT) [81].

Su et al. [82] demonstrated that fucoxanthin has a tremendous anti-inflammatory effect in a mouse sepsis model. LPS was used to induce sepsis in mice; when treated with 1 mg/kg (b.w.) of fucoxanthin, the survival rate can duplicate (20% to 40%). Fucoxanthin is related to the reduced levels of the pro-inflammatory cytokines’ TNF-α and IL-6 and the inhibition of the NF-ƘB inflammatory pathway [82].

Knowing the anti-inflammatory properties of fucoxanthin, Wu et al. [83] produced a nanofiber membrane named PLA/PEGDA-EDT@rGO-fucoxanthin (PPGF) that can capture ROS. Poly(ethyleneglycol)diacrylate(PEGDA)-1,2-ethanedithiol (EDT) copolymer (PEGDA-EDT) is responsible for the ROS capture, reduced graphene oxide (rGO) is the drug carrier, and fucoxanthin (**26**) attenuates osteoarthritis (OA) [83]. In response to hydrogen peroxide, the nanofiber membrane exhibited sustained and long-term fucoxanthin release behaviour in vitro (at least 66 days). Moreover, it showed low cytotoxicity and exceptional ability to capture ROS. PPGF showed excellent anti-inflammatory and antioxidant effects on IL-1β-induced chondrocytes by potent ROS scavenging; however, it is possible that its mechanism of action also involves the upregulation of antioxidative enzymes [83].

### 2.6. Fucosterol

**Figure 6:**
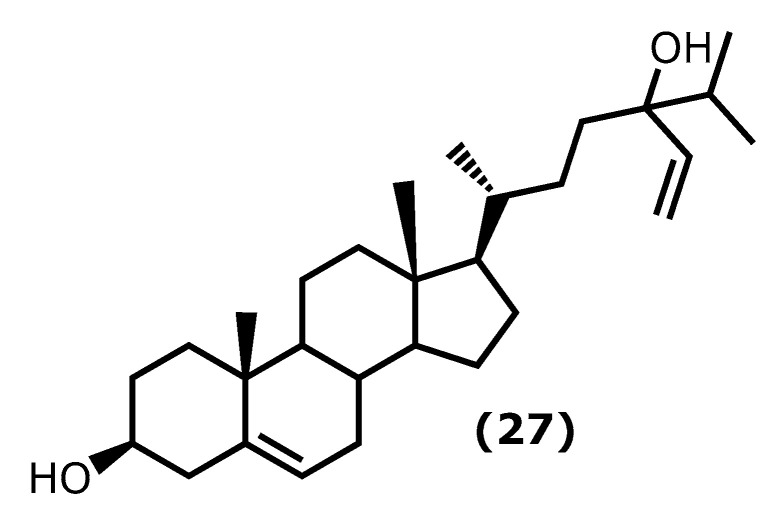
Fucosterol structure.

Fucosterol ((3*S*,10*R*,13*R*,17*R*)-17-((2*R*)-5-hydroxy-5-isopropylhept-6-en-2-yl)-10,13-dimethyl-2,3,4,7,8,9,10,11,12,13,14,15,16,17-tetradecahydro-1*H*-cyclopenta[*a*]phenanthren-3-ol (**27**)) (Figure 6) is one of the dominant sterols in marine macroalgae. Brown macroalgae contain higher levels of fucosterol (**27**) than green and red macroalgae. It can be found in brown macroalgae and isolated from species of the genera *Laminaria*, *Undaria*, *Sargassum*, and *Ecklonia* [84,85]. It is known to present several health benefits [86], and it is also known that fucosterol (**27**) has effects on several inflammatory pathways, such as decreasing the expression of p50 and p65 mRNA and the activity of NF-κB promoter in a dose-dependent manner, inhibiting the expression of TNF-α, COX-2, IL-1β, and IL-6 [87,88]. It also reduced the inflammatory response caused by solar ultraviolet radiation (UVR) [89].

More specific assays showed that fucosterol (**27**) protects LPS-induced acute lung injury (ALI) in mice [90]. The mechanism of action was revealed to be through the inhibition of TNF-α, IL-1β, and IL-6 levels in the bronchoalveolar lavage fluid (BALF) and the LPS-stimulated alveolar macrophages, reducing their expression by about 50%, when compared to the untreated group [90].

Sun et al. [91] demonstrated the protective mechanisms of fucosterol (**27**) on cobalt chloride (CoCl_2_)-induced hypoxia damage to keratinocytes (HaCaT). It attenuates CoCl_2_-induced excess expression of IL-6, IL-1β, and TNF-α and suppresses the phosphorylation of PI3K and Akt and the accumulation of HIF1-α simulated by CoCl_2_ [91]. On the other hand, Mo et al. [92] showed that (**27**) attenuated serum liver enzyme levels, hepatic necrosis, and apoptosis induced by TNF-α, IL-6, and IL-1β. It also showed the effect of this compound in the reduction in P38 MAPK, and NF-κB signalling was accompanied by PPARγ activation [92].

In the last years, Wong et al. [93] showed that (**27**) protects against amyloid β (Aβ)-mediated neuroinflammation by inhibiting the production of IL-6, IL-1β, TNF-α, NO, and PGE2 in LPS- or Aβ-induced microglial cells. Moreover, a similar study reported the fucosterol (**27**) effect on attenuate particulate matter CPM-induced inflammatory responses in A459 human lung epithelial cells through lowering the P65 and P50 nuclear translocation and the p38 mitogen-activated protein kinase (MAPK) phosphorylation, extracellular signal-regulated kinases 1/2 (ERK1/2) and c-Jun *N*-terminal kinases (JNK), and the levels of COX-2, PGE2, TNF-α, and IL-6 [94].

### 2.7. Caulerpin

**Figure 7:**
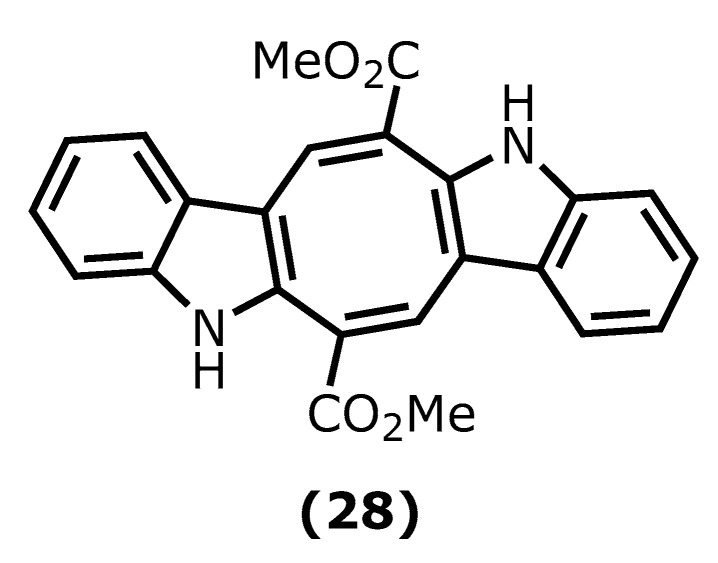
Caulerpin structure.

Caulerpin (dimethyl(6*E*,13*E*)-5,12-dihydrocycloocta [1,2-*b*:5,6-*b*′]diindole-6,13-dicarboxylate (**28**)) is an alkaloid found in seaweeds and presents desirable anti-inflammatory activity mainly attributed to indole moiety. The two indole units are linked together by a cyclooctane ring forming the 5,12-dihydrocycloocta [1,2-*b*:5,6-*b*′]diindole nucleus with two methoxycarbonyl groups at C-6 and C-13 (Figure 7) [95,96].

Caulerpin (**28**) has been isolated mainly from green and red algae species, such as *Caulerpa racemose* (Forsskål), J. Agardh, 1873, *Caulerpa sertularioide* (S. G. Gmelin), M. Howe, 1905, and *Caulerpa mexicana*, Sonder ex Kützing, 1849, and its in vivo anti-inflammatory activity has been investigated. For instance, its potency against ear oedema and peritonitis in mice induced by capsaicin (8-methyl-*N*-vanillyl-6-nonenamide) and carrageenan was assessed. Caulerpin (**28**) caused a significant reduction in plasma extravasation of mice ears (55.8%), when compared to capsaicin and leukocyte reduction [97].

Lucenna et al. [98] also reported the caulerpin (**28**) anti-inflammatory effect on the murine model of peritonitis and ulcerative colitis. The authors established that caulerpin (**28**) at 4 mg/kg triggered improvement of the Disease Activity Index (DAI) and attenuated the colon shortening and damage. This dose reduced the TNF-α, IFN-γ, IL-6, IL-17, and NFκB p65 levels and increased the levels of IL-10 in the colon tissue [98].

### 2.8. Fatty Acids

Fatty acids (FAs) are classified according to their carbon-chain length and sometimes the number of double bonds present. Long-chain fatty acids should have more than twelve carbons in the chain, whereas very long-chain should contain more than twenty-two. In the case of the polyunsaturated fatty acids, a further classification of omega-3 (ω-3) and omega-6 (ω-6), based on the position of the first double bond on the methyl terminal end, can be found in the literature. Polyunsaturated fatty acids (PUFAs) are known to play a vital role in body homeostasis. In general, higher levels of ω-6 polyunsaturated fatty acids are associated with constriction of blood vessels, inflammation, and platelet aggregation, whereas ω-3 may help to resolve inflammation and alter the function of vascular biomarkers [99]. It is known that ω-3 PUFAS has an important role in the reduction of depressive symptoms and exerts an anti-inflammatory action by the production of distinct metabolites, such as resolvins D (RvD) and E series, and maresins (MaR) and protectins (PD). The *Z*-4,7,10,13,16,19-docosahexaenoic acid (DHA)-derived trihydroxydocosahexanoic acid mediators termed RvD are produced by a series of reactions involving COX-2 and 5-LOX or by a pathway involving lipoxygenase enzymes and other reactions. The metabolism of DHA initially occurs by 15-lipoxygenase and then a series of other reactions generates a dihydroxy derivative termed protectin D1. The trihydroxyeicosapentaenoic acid mediators, termed RvE, form from *Z*-5,8,11,14,17- eicosapentaenoic acid (EPA) by a similar series of reactions involving COX-2 and 5-LOX [100]. These mediators appear to act as a potent anti-inflammatory in psychiatric, neurodegenerative, and neurological diseases. On a cellular level and in a depression model, RvDs increased serotonin levels; on the other hand, they decreased gliosis in neurodegenerative disorders. Protectins prevented neurite and dendrite retraction and apoptosis in models of neurodegeneration, whereas maresins reduced cell death [101].

**Figure 8:**
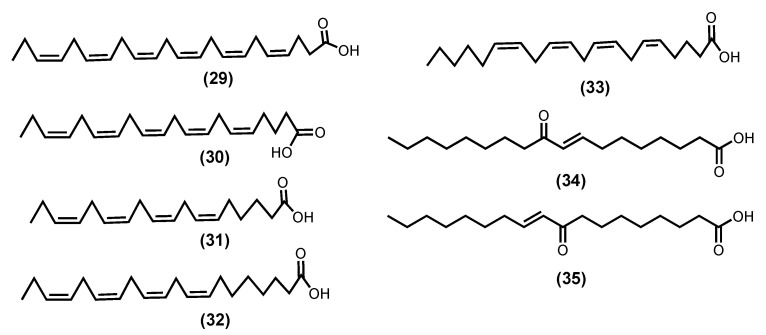
Fatty acids structures.

Palmitic acid, a saturated fatty acid, is the most prevalent in seaweeds; nevertheless, ω-6 and ω-3 PUFAs [99], such as DHA (**29**), EPA (**30**), Z-6,9,12,15-octadecatetraenoic acid (stearidonic acid-SA) (**31**), Z-8,11,14,17-eicosatrienoic acid (ETA) (**32**), and Z-5,8,11,14-icosa-5,8,11,14-tetraenoic acid (arachidonic acid-AA) (**33**) (Figure 8), are also commonly isolated from seaweeds [99,102]. There is evidence that these molecules can play a key role in the inflammation process [100,103]. Pro-inflammatory PGE2 and leukotriene B4 (LTB4) are produced during the metabolism of AA (**33**) through COX and 5-lipoxygenase of leukotriene-A4 (LTA4) hydrolase enzymatic pathway, respectively. At the same time, DHA (**29**) and EPA (**30**) compete with AA (**33**) metabolism, thus reducing the production of PGE2 and LTB4. The metabolism of ETA (**32**) by 5-lipoxygenase form leukotriene-A3 (LTA3) inhibits the LTA4 hydrolase necessary for the production of LTB4, thus acting as an anti-inflammatory by inhibiting LTB4 production [104,105]. Moreover, SA (**31**) and EPA (**30**), which were extracted from *Undaria pinnatifida* (Harvey), Suringar, 1873, exhibited anti-inflammatory activity against mouse ear enema, erythema, and blood flow induced by phorbol myristate acetate. Whereas SA (**31**) extracted from *Ulva pertusa*, Kjellman, 1897, was able to suppress the production of LTB4, Leukotriene C4 (LTC4), and 5-hydroxyeicosatetraenoic acid (5-HETE) in an MC/9 mouse mast cell [99,100,101,102,103,104,105].

Recently, in a randomized, controlled, double-blind, and crossover study, 21 subjects (9 men and 12 postmenopausal women) with chronic inflammation and some characteristics of metabolic syndrome received a 10-week supplementation with EPA (**30**) or DHA (**29**) (3 g/day), relative to a 4-week lead-in phase of high oleic acid sunflower oil (3 g/day, defined as baseline). This study showed that both EPA (**30**) and DHA (**29**) significantly lowered the tricarboxylic acid (TCA) cycle intermediates, the interconversion of pentose and glucuronate, alanine, aspartate, and glutamate pathways (FDR < 0.05), and that DHA (**29**) had a greater effect on the TCA cycle than EPA (**30**). This study demonstrated a significant impact of both compounds on the cell metabolism of individuals with chronic inflammation [106].

Our final choice relay on two enones (*E*)-9-oxooctadec-10-enoic acid (**34**) and (*E*)-10-oxooctadec-8-enoic acid (**35**) fatty acids isolated from *Gracilaria verrucose* (Hudson) Papenfuss, nom. rejic., 1950, (the current accepted name is Gracilariopsis longissimi (S. G. Gmelin) Steentoft, L. M. Irvine and Farnham, 1995), and for which the inhibition of the production of the inflammatory markers’ nitric oxide, TNF-α, and IL-6, in a dose-dependent manner and LPS-stimulated RAW264.7 cells, was reported. They suppressed NF-κB reporter activity by interfering with the nuclear translocation of NF-κB and suppressed JAK/STAT (p-STAT1) signalling [107].

## 3. Macroalgae Commercially Available Products

Early seaweed was mainly collected in its natural form; however, wild seaweed resources are limited with the constantly growing market for the food industry, medical and cosmetic uses, and energy sources. Alternatives pass to seaweed culture on land, sea, desert, and even in integrated aquaculture systems. The *Financial Times* has reported that the global population will rise to 10 billion by 2050. Furthermore, algae could supply the protein needed for people while conserving natural resources. It can be part of the solution by providing an excellent alternative to traditional crops as they do not require arable land and can grow on minimal nutrients [108]. The global seaweed market size was, in 2017, around USD 4097.93 million, and it is projected to reach USD 9075.65 million by 2024, registering a compound annual growth rate (CAGR) of 12.0% from 2018 to 2024 [109,110]. So, it is clear that seaweeds have emerged as one of the most promising resources due to their remarkable adaptability, short development period, and resource sustainability. The advantages of using seaweed in the food, cosmetic, and medical fields are huge in terms of economics and sustainability.

The European Algae Biomass Association (EABA) was founded in 2009 and, from the beginning of its formation, started to promote synergies between academia, industry, and decision-makers, aiming to establish the algae industry. Since the EABA’s creation, some EU countries have created their associations; the Fédération des Spiruliniers in France and PROALGA (Associação Portuguesa de Produtores de Algas) in Portugal are excellent examples. Currently, around 420 companies from 23 countries produce 36% of seaweed in Europe [111]. As a result, algae or algae products are nowadays used, usually in the EU, as food or food ingredients. For instance, cookies, pasta, bread, and beverages, that are produced using algae are increasing in the European market, holding a 1.34% share of the new European foods and drinks launched in 2017 [112,113].

Algae are considered appreciated components in the medical field, particularly algal hydrogels and hydrocolloids, which are actually polysaccharide-based hydrogels. These algal hydrogels are widely used in wound healing, drug delivery, in vitro cell culture, and tissue engineering. From the structural point of view, these gels are similar to the tissues’ extracellular matrix and can be manipulated to perform several vital roles. Some drug-specific gels have been clinically used for wound healing and have proven efficient and safe. In fact, wound healing and drug delivery applications are excellent examples of continuous and sequential drug release [114]. So, the development of new products with this specificity is vital in the future. One example is ACTIVHEAL^®^ ALGINATE, which is already used in medical treatment [115].

Cosmetics is a field in which seaweed has consolidated its use. A wide variety of products, from slimming creams to perfumes, shampoos, sunscreens, and bath salts, can be found on the market. In terms of seaweed-based products on the market, Revertime™, Sealgae^®^, Codiavelane^®^, Algowhite^®^, Pheofiltrat^®^, and Actiseane^®^ are some examples of products and trademarks, most of them used in cosmetics, algotheraphy, and thalassotherapy [116].

Although the products mentioned above obtained from macroalgae have exciting applications, with some possible anti-inflammatory ones [114], it is evident that the isolated compounds’ applications are far from being commercially available. This is primarily due to the lack of proper toxicological assays and clinical trials.

## 4. Conclusions

The analysis of the macroalgae specialized metabolites’ anti-inflammatory potential, herein presented and discussed, shows that the attributes commonly known for these marine species may be due to their specialized metabolites. In particular, the established anti-inflammatory activities for macroalgae [23,85] must be owing to some of the metabolites mentioned above. Moreover, these metabolites may explain some use of macroalgae in the production of biomaterials [117]. The chosen specialized metabolites present exciting activities, in some cases lowering concentrations and having different biological targets (Table 1), which make them suitable leader compounds for developing new anti-inflammatory drugs. Moreover, some compounds were also tested in in vivo assays and maintained their activity (Table 1). Naturally, toxicological assays and clinical trials are essential to establish the compound’s potential. In this regard, it is worth mentioning the more studied compounds, such as fucoxanthin (**26**), fucosterol (**27**), and caulerpin (**28**), for which clinical trials are needed. Nevertheless, we hope this survey will incentivize future investigations concerning the specialized metabolites herein discussed and the search for other bioactive compounds isolated from macroalgae.

In our final comments, we highlight that the isolation of these bioactive is still problematic and prevent their industrial use. Mostly, they are costly procedures and allow for only small amounts of pure compounds, so using all macroalgae is still more economical.

The old traditional technique of solid–liquid extraction is still the most employed because it is easier to use and less expensive. However, it also involves more energy consumption and the use of less environmentally friendly solvents. The use of non-conventional extraction methods is highly recommended. In this regard, several authors are investigating alternative methods, such as microwave-assisted extraction, ultrasonic-assisted extraction, pressurized solvent extraction, supercritical fluid extraction, and enzyme-assisted extraction [118,119,120,121,122,123,124], to obtain the bioactive compounds in more environmentally friendly conditions. Some of these methods still need extra optimizations to incentive their use.

Note: The macroalgae full names are accordingly the Algaebase (https://www.algaebase.org/) and were confirmed on 18 December 2022.

**Table 1:** Resume of the main anti-inflammatory potential disclosed for seaweeds specialized metabolites.

Compound (Number)	Source *	Concentration Tested	Experimental Model	Pharmacological Markers
Phloroglucinol (**1**) ^1^ [40]	*Ecklonia cava*	10 μΜ	RAW 264.7 cells HT1080 cells	↓ TNF-α, IL-1β e IL-6, PGE_2_ Inhibit MMP-2 and MMP-9
Dieckol (**2**) ^2^ [42,47]	*Eisenia* sp.	10 and 20 μΜ	HUVECs Mice treated by high mobility group box 1 protein (HMGB1)	↓ LPS-mediated hyperpermeability (74.9%) ↓ LPS-induced HMGB1 release ↓ acetic acid induced-hyperpermeability and carboxymethylcellulose-induced leukocytes migration (55%)
Eckol (**3**) ^3^ [43]	*Eisenia* sp. *Eckonia* sp.	1–10 μΜ	*Propionibacterium acnes* induced HaCaT cells	↓ TNF-α ↓ COX-2, iNOS
Phlorofucofuroeckol B ^4^ (**4a**) [45]	*Eisenia arborea*	75 μΜ	ICR strain mouse	inhibition of ear edema induced by AA (42.2%), by TPA (38.4%), and by OXA (41.0%). EGCG inhibits 12.9%, 13.8%, and 5.7% of ear edema induced by AA, TPA, and OXA, respectively
Phlorofucofuroeckol A ^5^ (**4b**) [41,45]	*Eisenia arborea* *Ecklonia stolonifera* Okamura 1913 ^a^	40 μΜ 10 μΜ 75 μΜ	LPS-stimulated BV-2 cells RBL-2H3 cells ICR strain mouse	↓ TNF-α, IL-1β e IL-6 ↓ COX-2, NO ↓ phosphorylation Akt, ERK, JNK ↓ histamine, leukotriene B4, PEG_2_ inhibition of ear edema induced by AA (30.5%), TPA (31.7%), and OXA (23.4%). EGCG inhibits 12.9%, 13.8%, and 5.7% of by AA, TPA, and OXA, respectively
Fucofuroeckol-A (**5**) ^6^ [39]	*Eisenia bicyclis* (Kjellman) Setchell 1905	1–100 μΜ	LPS-induced RAW 264.7 cells	↓ NO, PGE_2_, iNOS ↓ TNF-α, IL-1β, IL-6 ↓ COX-2
6,6′-Bieckol (**6**) ^7^ [44,45]	*Ecklonia cava* *Eisenia arborea*	100 and 200 μΜ 75 μΜ	LPS-induced RAW 264.7 cells ICR strain mouse	↓ NO, PGE_2_, iNOS ↓ TNF-α, IL-6 ↓ COX-2 inhibition of ear oedema induced by AA (41.9%), TPA (34.2%), and OXA (17.8%). EGCG inhibits 12.9%, 13.8%, and 5.7% of by AA, TPA, and OXA, respectively
6,8′-Bieckol (**7**) ^8^ [45]	*Eisenia arborea*	10 μΜ 75 μΜ	RBL-2H3 cells ICR strain mouse	↓ COX-2 mRNA expression inhibition of ear oedema induced by AA (39.8%), TPA (49.4%), and OXA (77.8%). EGCG inhibits 12.9%, 13.8%, and 5.7% of by AA, TPA, and OXA, respectively
8,8′-Bieckol (**8**) ^9^ [45]	*Eisenia arborea*	10 μΜ 75 μΜ	RBL-2H3 cells ICR strain mouse	↓ histamine, leukotriene B4, PEG_2_ inhibition of ear oedema induced by AA (21.0%), TPA (31.7%), and OXA (32.3%). EGCG inhibits 12.9%, 13.8%, and 5.7% of by AA, TPA, and OXA, respectively
Octaphlorethol A (**9**) ^10^ [46]	*Ishige foliacea*	6.2 and 12.5 μΜ	CpG-stimulated BMCD and BMDM	↓ TNF-α, IL-6, IL12 p40
Vidalol A (**10**) ^11^ [52]	*Vidalia obtusiloba*	n. r.	phorbol ester (PMA)—induced swelling of the mouse ear Enzymatic activity	↓ eodema (58–82%) ↓ phospholipase A_2_
Vidalol B (**11**)^12^ [52]	*Vidalia obtusiloba*	n. r.	phorbol ester (PMA)—induced swelling of the mouse ear Enzymatic activity	↓ eodema (58–82%) ↓ phospholipase A_2_
3-BDB (**12**) ^13^ [53,54]	*Polysiphonia morrowii* *Polysiphonia urceolata* *Rhodomela confervoides*	12.5, 25, 50, and 100 μM 100 mg/kg	LPS-stimulated RAW 264.7 BALB/c mice induced by DNCB	↓ IL-6, phosphorylation NF-ΚB ↓ STAT1; Tyr 701 ↓ edema inflammation, AD symptoms, Ig_2_
BEMB (**13**) ^14^ [56]	*Polysiphonia morrowii*	12.5–50 μM	LPS-stimulated RAW 264.7 and zebrafish embryos	↓ NO, iNOS, COX-2, NF-ƘB
BBDE (**14**) ^15^ [57]	*Polysiphonia morrowii*	0.1, 1, 2 μM	LPS-stimulated RAW 264.7	↓ NO, iNOS, COX-2, PGE_2_, TNF-α, IL-6, IL-1β
Compound (**15**) ^16^ [58]	*Gracilaria opuntia*	n. r.	Enzymatic activity	↓ COX-2, 5-LOX
Compound (**16**) ^17^ [59]	*Gracilaria opuntia*	n. r.	Enzymatic activity	↓ 5-LOX
Compound (**17**) ^18^ [59]	*Gracilaria opuntia*	n. r.	Enzymatic activity	↓ 5-LOX
Compound (**18**) ^19^ [60]	*Gracilaria salicornia*	n. r.	Enzymatic activity	↓ COX-2, 5-LOX
Compound (**19**) ^20^ [60]	*Gracilaria salicornia*	n. r.	Enzymatic activity	↓ COX-2, 5-LOX
Rutin (**20**) ^21^ [61]	*Porphyra dentata*	80–250 μM	LPS-stimulated RAW 264.7	↓ NO, iNOS, NF-ƘB
5β-Hydroxypalisadin B ^22^ (**21**) [64]	*Laurencia snackeyi*	50 μM 0.25, 0.1 and 1 µg/mL	LPS-induced RAW 264.7 LPS-induced zebrafish embryo	↓ NO, COX-2, iNOS ↓ NO, Improved survival, heart rate and yolk sac oedema size
Neorogioltriol (**22**)^23^ [66,67]	*Laurencia glandulifera*	8 μM 1 mg/kg	LPS-induced RAW 264.7 Writhing test in mice Formalin test in rats	↓ NO, iNOS ↓ macrophage activation induce Arginase 1, MRC1, miRNA miR-146a ↓ writhing response induced by acetic acid by 88.9% ↓ 2° phase formalin test in 48.7%
Neorogioldiol (**23**) ^24^ [67]	*Laurencia* sp	62.5 μM	LPS-induced RAW 264.7 C57BL/6 mice	↓ NO, iNOS ↓ macrophage activation induce Arginase 1, MRC1, miRNA miR-146a ↓ tissue damage, TNF-α, IL-6, IL-12
Compound (**24**) ^25^ [67]	*Laurencia* sp	10 μM	LPS-induced RAW 264.7 C57BL/6 mice	↓ NO, iNOS ↓ macrophage activation induce Arginase 1, MRC1, miRNA miR-146a ↓ tissue damage, TNF-α, IL-6, IL-12
Compound (**25**) ^26^ [68]	*Gracilaria Salicornia*	n. r.	Enzymatic activity	↓ 5-LOX
Fucoxanthin (**26**) ^27^ [74,77,82]	*Sargassum siliquastrum* (Mertens ex Turner) C.Agardh 1820	0.1–1 mg/kg 15, 30, 60 μM	LPS-induced sepsis in mice LPS-induced RAW 264.7 LPS-activated BV-2 microglia	↓ TNF-α, IL-6, IL-12, NF-ƘB ↑ rate of survival ↓ iNOS, COX-2, mRNA, TNF-α, IL-6 ↓ iNOS, COX-2, mRNA, TNF-α, IL-6 ↓ Akt, NF-Κb, ERK, p38 MAPK
Fucosterol (**27**) ^28^ [90,91,93]	*Undaria pinnatifida* *Hizikia fusiformis* (Harvey) Okamura 1932 ^b^ *Panida australis* ^c^	15, 30, 60 mg/kg 1–10 µM 0.004, 0.2, 10 µM	LPS-induced ALI in mise CoCl_2_-induced hypoxia in keratinocytes LPS or Aβ-induced BV2 (microglial) cells	↓ lung histopathologic changes, wet-to-dry ratio ↓ TNF-α, IL-6, IL-1β, NF-κB ↓ IL-6, IL-1β, TNF-α, pPI3K and pAkt and HIF1-α accumulation ↓ L-6, IL-1β, TNF-α, NO, PGE_2_
Caulerpin (**28**) ^29^ [97,98]	*Caulerpa racemosa* (Forsskål) J.Agardh 1873 *Caulerpa sertularioide*	100 μmol/kg 4 mg/kg	Swiss albino mice C57BL/6 mice with colitis induced DSS	↓ formalin effects in both phases by 35.4% and 45.6%. reduction 55.8% on capsaicin-induced ear oedema model ↓ recruit cells (48.3%) on carrageenan-induced peritonitis triggering improvement of DAI and attenuating the colon shortening/ damage ↓ TNF-α, IFN-γ, IL-6, IL-17, NFκB p65 ↑ IL-10 in the colon tissue
*Z*-4,7,10,13,16,19-Docosahexaenoic acid-DHA (**29**) [102,104,106]	*Sargassum natans* (Linnaeus) Gaillon 1828	n. r. 3 g/day	21 volunteers (9 men and 12 postmenopausal women) with chronic inflammation and some characteristics of metabolic syndrome	RvE1 protect tissue counterregulates pro-inflammatory gene expression ↓ fumarate, pyruvate, citrate, isocitrate, malate, α-ketoglutarate ↑ succinate, glucuronate
*Z*-5,8,11,14,17-Eicosapentaenoic acid-EPA (**30**) [99,104,106]	*Vertebrata lanosa* (Linnaeus) T.A.Christensen 1967 *Palmaria palmata* (Linnaeus) F.Weber and D.Mohr 1805 *Laminaria digitata* (Hudson) J.V.Lamouroux 1813	n. r. 3 g/day	21 volunteers (9 men and 12 postmenopausal women) with chronic inflammation and some characteristics of metabolic syndrome	RvE1 protect tissue counterregulates pro-inflammatory gene expression ↓ fumarate, α-ketoglutarate ↑ UDP-glucuronate, glucuronate
*E*-9-Oxooctadec-10-enoic acid (**34**) [105]	*Gracilaria verrucose* (Hudson) Papenfuss, nom. Rejic. 1950 ^d^	50–100 μM	LPS-induced RAW 264.7	↓ NO, TNF-α, IL-6 ↓ NF-ƘB, JAK/STAT
*E*-10-Oxooctadec-8-enoic acid (**35**) [105]	*Gracilaria verrucose* ^d^	50–100 μM	LPS-induced RAW 264.7	↓ NO, TNF-α, IL-6 ↓ NF-ƘB, JAK/STAT

* The macroalgae full names are accordingly the Algaebase (https://www.algaebase.org/ (accessed on 18 December 2022)); ^1^ benzene-1,3,5-triol; ^2^ 4-(4-((6-(3,5-dihydroxyphenoxy)-4,7,9-trihydroxydibenzo[*b*,*e*][1,4]dioxin-2-yl)oxy)-3,5-dihydroxyphenoxy)dibenzo[*b*,*e*][1,4]dioxine-1,3,6,8-tetraol; ^3^ 4-(3,5-dihydroxyphenoxy)dibenzo[*b*,*e*][1,4]dioxine-1,3,6,8-tetraol; ^4^ 4,9-bis(3,5-dihydroxyphenoxy)benzo[*b*]benzo[5,6][1,4]dioxino[2,3-*e*]benzofuran-1,3,6,10,12-pentaol; ^5^ 4-(3,5-dihydroxy-phenoxy)benzo[*b*]benzo[5,6][1,4]dioxino[2,3-*e*]benzofuran-1,3,6,9,10,12-hexaol; ^6^ 4-(3,5-dihydroxyphenoxy)benzo[*b*]benzo[5,6][1,4]dioxino[2,3-*e*]benzofuran-1,3,6,9,10,12-hexaol; ^7^ 6,6′-bis(3,5-dihydroxyphenoxy)-[1,1′-bidibenzo[*b*,*e*][1,4]dioxin]-2,2′,4,4′,7,7′,9,9′-octaol; ^8^ 6,9′-bis(3,5-dihydroxyphenoxy)-[1,2′-bidibenzo[*b*,*e*][1,4]dioxin]-1′,2,3′,4,6′,7,8′,9-octaol; ^9^ 9,9′-bis(3,5-dihydroxyphenoxy)-[2,2′-bidibenzo[*b*,*e*][1,4]dioxin]-1,1′,3,3′,6,6′,8,8′-octaol; ^10^ 2-(4-(4-(4-(4-(4-(4-(3,5-dihydroxyphenoxy)-3,5-dihydroxyphenoxy)-3,5-dihydroxyphenoxy)-3,5-dihydroxyphenoxy)-2,6-dihydroxyphenoxy)-2,6-dihydroxyphenoxy)-2,6-dihydroxyphenoxy)benzene-1,3,5-triol; ^11^ 2-bromo-4-(2,3-dibromo-4,5-dihydroxybenzyl)benzene-1,3,5-triol; ^12^ 2-bromo-4,6-bis(2,3-dibromo-4,5-dihydroxybenzyl)benzene-1,3,5-triol; ^13^ 3-bromo-4,5-dihydroxybenzaldehyde; ^14^ 3-bromo-5-(ethoxymethyl)benzene-1,2-diol; ^15^ bis(3-bromo-4,5-dihydroxybenzyl)ether; ^16^ 2-acetoxy-2-(5-acetoxy-4-methyl-2-oxotetrahydro-2*H*-pyran-4-yl)ethyl 4-(3-methoxy-2(methoxymethyl)-7-ethyl-3,4,4a,7,8,8a-hexahydro-2*H*-chromen-4-yloxy)-5-methylheptanoate; ^17^ 5-[7-(5-ethyl-3,4-dimethoxycyclooctyl)benzofuran-6-yl]-7-methyl-3,4,7,8-tetrahydro-2*H*-oxocin-2-one; ^18^ 2-(3-ethyl-9-(2-methoxyethoxy)-1-oxo-2,3,4,9-tetrahydro-1*H*-xanthen-2-yl)ethyl 5-hydroxy-9-methoxy-7,8-dimethyl-8-(5-methylfuran-2-yl)nona-3,6-dienoate; ^19^ 4′-[10′-[7-hydroxy-2,8-dimethyl-6-(pentyloxy)-2*H*-chromen-2-yl]ethyl]-3′,4′-dimethyl-cyclohexanone; ^20^ 3′-[10′-(8-hydroxy-5-methoxy-2,6,7-trimethyl-2*H*-chromen2-yl)ethyl]-3′-methyl-2′-methylene cyclohexyl butyrate; ^21^ 2-(3,4-dihydroxyphenyl)-5,7-dihydroxy-3-(((2*S*,4*S*,5*S*,6*R*)-3,4,5-trihydroxy-6-((((2*R*,3*R*,4*R*,5*R*,6*S*)-3,4,5-trihydroxy-6-methyltetrahydro-2*H*-pyran-2-yl)oxy)methyl)tetrahydro-2*H*-pyran-2-yl)oxy)-4*H*-chromen-4-one; ^22^ (2*R*,5*R*,7*S*,9a*S*)-7-bromo-2-(bromomethyl)-3,6,6,9a-tetramethyl-2,5,5a,6,7,8,9,9a-octahydrobenzo[*b*]oxepin-5-ol; ^23^ (1*R*,5*S*,6*S*)-5-(1-((3*R*,4*S*)-3-bromo-4-hydroxy-4-methylcyclohexyl)vinyl)-1,4,4-trimethyloctahydropentalene-1,6-diol; ^24^ (1*R*,6*S*)-6-bromo-5-(1-((3*R*,4*S*)-3-bromo-4-hydroxy-4-methylcyclohexyl)vinyl)-1,4,4-trimethyloctahydropentalen-1-ol; ^25^ *O*^11^,15-cyclo-14-bromo-14,15-dihydrorogiol-3,11-diol; ^26^ methyl 16(13→14)-abeo-7-labdebe-(12-oxo)carboxylate; ^27^ (3*R*)-3-hydroxy-4-((3*E*,5*E*,7*E*,9*E*,11*E*,13*E*,15*E*)-18-((1*S*,4*S*,6*R*)-4-hydroxy-2,2,6-trimethyl-7-oxabicyclo[4.1.0]heptan-1-yl)-3,7,12,16-tetramethyl-17-oxooctadeca-1,3,5,7,9,11,13,15-octaen-1-ylidene)-3,5,5-trimethylcyclohexyl acetate; ^28^ (3*S*,10*R*,13*R*,17*R*)-17-((2*R*)-5-hydroxy-5-isopropylhept-6-en-2-yl)-10,13-dimethyl-2,3,4,7,8,9,10,11,12,13,14,15,16,17-tetradecahydro-1*H*-cyclopenta[*a*]phenanthren-3-ol; ^29^ dimethyl(6*E*,13*E*)-5,12-dihydrocycloocta[1,2-*b*:5,6-*b’*]diindole-6,13-dicarboxylate; ^a^ Current accepted name *Ecklonia cava* subsp. *stolonifera* (Okamura), S. Akita, K. Hashimoto, T. Hanyuda and H. Kawai, 2020; ^b^ Current accepted name *Sargassum fusiforme* (Harvey) Setchell 1931; ^c^ Unknown name, others authors indicate *Posidonia australis* Hooker F., 1858; ^d^ Current accepted name *Gracilaripsis longissimi* (S. G. Gmelin) Steentoft, L. M. Irvine and Farnham, 1995; n. r. = non revealed.

## Data Availability

Not applicable.
